# An Exploratory Study Investigating the Prevalence of Gastrointestinal Symptoms in Collegiate Division I American Football Athletes

**DOI:** 10.3390/ijerph20156453

**Published:** 2023-07-27

**Authors:** Floris C. Wardenaar, Kinta D. Schott, Alex E. Mohr, Carmen P. Ortega-Santos, John E. Connolly

**Affiliations:** 1 College of Health Solutions, Arizona State University, Phoenix, AZ 85004, USA; kschott3@asu.edu (K.D.S.); aemohr@asu.edu (A.E.M.); 2 Division of Endocrinology, Metabolism, and Diabetes, Department of Medicine, University of Colorado Anschutz Medical Campus, Aurora, CO 80045, USA; carmen.ortega-santos@cuanschutz.edu; 3 Sun Devil Athletics, Arizona State University, Tempe, AZ 85287, USA; joeconnolly@asu.edu

**Keywords:** gastrointestinal symptom rating scale, protein use, gut, team-sport athletes, high-intensity exercise

## Abstract

Gastrointestinal (GI) symptoms may limit performance, but their prevalence and impact among team sports athletes is not well-documented. The objective of this study was to examine the prevalence of GI symptoms in a small sample of collegiate DI American football athletes, using a survey including the Gastrointestinal Symptoms Ratings Scale (GSRS). Forty-six athletes responded to the survey and reported scores for the 15-question GSRS with additional questions about dietary habits and supplement use. A total of 44 athletes were included in the study (45% of the current roster, age: 20.7 ± 1.7 years, 50% Afro-American or black, 39% skill position, 18% NSAIDs use, and 41% reporting protein supplement use); approximately half of the athletes (52%) reported experiencing GI complaints during exercise. Two-thirds of the athletes (61%) reported at least one or more GI symptoms in general, and 50% reported at least four moderate complaints. Seven athletes (16%) reported ≥2 severe GI symptoms with 5–13 moderate complaints. The most reported symptom was stomach pain (39%, n = 17), followed by hunger pain (36%, n = 16). Athletes reporting the use of protein supplements reported a higher GSRS score (22.0 and interquartile range (IQR) 17.0–31.8) vs. athletes not reporting protein use (15.0 and IQR 15.0–19.3), *p* = 0.001. Most athletes surveyed reported experiencing GI symptoms. A small group of these athletes reported multiple, varied, and severe symptoms that were associated with self-reported protein supplement use. In conclusion, the number of complaints varied among athletes, confirming the value of integrating the GSRS for screening purposes, and the expected need for individual dietary treatment approaches.

## 1. Introduction

There is strong evidence that gastrointestinal (GI) symptoms are common and problematic in endurance athletes, but research examining this subject among team-sport athletes is scarce [1]. Between 30–90% of athletes suffer GI symptoms, including bloating, constipation, and diarrhea, especially prominent in extensive aerobic activity [2], and increasing with exercise intensity [3,4]. These complaints may be multifactorial and result from impaired gut barrier function, local inflammation, microbiota disbalance, and/or stress-related nutrient malabsorption [2]. Most studies focus on GI distress during exercise, with a prevalence ranging from 4–96% [2]; some have focused on GI complaints directly following endurance exercise, with a prevalence up to 50% [5,6,7]; however, little is known about general GI complaints that athletes experience during normal life outside of their sports activities [8], especially in team sports athletes [1]. For example, American football athletes, who place their bodies under extreme stress during training in preparation for the regular season, may experience GI distress which, even in the absence of pathologies, may lead to GI distress that impairs wellbeing and performance.

Studies specific to team sports are needed, because these activities are inherently different from endurance activities. Specifically, American football involves repeated accelerations–decelerations and high-intensity exercise [9,10], and due to the nature of the sport, the preparation for the competitive season often starts in mid-summer, resulting in an extra environmental burden on the players [11]. In addition, the frequent physical contact between players could in theory contribute to GI disturbances through mechanical trauma as well [1,12]. The specific nature of the American football game, including players with a wide variety of body sizes and body compositions, demands more specific insight into the GI symptoms players experience. In addition to various positional differences and body compositional differences, race-based differences may play an additional role in the occurrence of GI symptoms in these athletes. In 2022, the largest cohort of participating American football athletes at the division I level were reported as black or Afro-American (48%, n = 14,709) [13]. Those of African descent are reported to have the third greatest lactase non-persistence (75%) which is the most common genetically related cause of lactose intolerance [14]. Commonly, the best practice to avoid the symptoms relating to this intolerance is the avoidance of lactose-containing foods [1,15].

The development of GI complaints, as well as their mitigation, have been associated with dietary factors such as pre-race meal timing and food, including the consumption of high fiber, fat, and protein diets [1]. In addition, nutritional supplements such as probiotics may impact gut health [2,16]. Nutritional supplementation is a common occurrence in collegiate athletics. In a study by Vento and Wardenaar, 32% of the athletes reported the use of probiotic-rich foods or probiotic supplements [17]. Despite the popularity of pre- and probiotics, no clear impact of these substances has been shown in relieving gastrointestinal symptoms at rest [16]. Whey protein may provide many benefits to the body, including muscular protein synthesis, antimicrobial activity, and immune system benefits [18], but it also has been anecdotally linked to the onset of GI complaints when consumed in large amounts after practice. The use of protein supplements in athletes is widespread, including protein shake usage (66%) and protein bars (60%) [17]. Finally, creatine, which is used by a majority of collegiate athletes (55%), is reported to be the second most common ergogenic aid behind caffeine (63%), to improve performance [17]. Creatine reportedly induces various GI symptoms, including stomach cramps, diarrhea, and nausea, with dosage levels equal to or greater than 10 g/serving [19]. Aside from dietary factors, the use of non-steroidal anti-inflammatory drugs (NSAIDs) is common in athletes to relieve soreness and pain resulting from participation in training and competition [20]. The use of NSAIDs causes the inhibition of the COX-1 pathway which, when expressed, helps to maintain gastric mucus secretions [21]. These mucus secretions help to protect the lining of the stomach and intestinal walls, and prevent damage from gastric juices and opportunistic bacterial growth (i.e., pathogens that do not usually infect healthy hosts but may produce infections in immunocompromised persons), which may increase the risk of GI discomfort [22]. Although NSAIDs have been shown to be effective and safe, chronic and over-use of NSAIDs can lead to an increased risk of GI hemorrhages and ulcers [23]. A total of 62% of American football athletes reported the use of NSAIDs regularly to alleviate soreness after a tough workout or competition [20], while 50% of all athletes surveyed felt they overused these anti-inflammatory drugs [20].

The effects of intense endurance exercise on GI distress have been extensively researched [24], and it is likely that hot conditions increase the prevalence of GI complaints [25]. However, little is known about the effects of team-based high-intensity interval sports like American football on GI symptoms. The aim of this study was to reach a better exploratory understanding of the self-reported prevalence of GI symptoms and their severity at an NCAA Division I level American football team operating in the hot and dry U.S. Southwest. To assess potential factors related to GI distress, we performed multiple stratifications to investigate the relationship between GSRS, race and ethnicity, playing position, food allergies, the use of the most frequently reported nutritional supplements, and NSAIDs use. We hypothesized that black or Afro-American athletes, due to their higher prevalence of lactose intolerance [14], or those who were considered “size” athletes [26], would be at an increased risk for higher gastrointestinal symptom rating scores. As the use of NSAIDs and nutritional supplements has previously been linked with GI distress [27,28,29], we also investigated if their use was associated with self-reported GI symptoms.

## 2. Materials and Methods

### 2.1. Study Design and Population

This small explorative cohort study in a non-probability sample of collegiate American football athletes aimed to gather more information about the GI symptoms they experience during their preparation for the regular competition season. For this purpose, athletes were approached at the football dining facility and the football nutrition bar at lunch time after their strength and conditioning practice. The athletes were given a handwritten questionnaire which initially included informed consent that had to be completed immediately, and then returned prior to exiting the facility. No direct incentives were offered; however, athletes were informed that the information gained from this study may help with athletic department policymaking. The results of the questionnaire, which included the Gastrointestinal Symptom Rating Scale (GSRS), were analyzed for general outcomes and stratifications for factors that may be associated with the prevalence of GI symptoms, such as race, playing position, nutritional supplement use, and the use of NSAIDs.

For this study, we approached all football players that ordered lunch during a consecutive Friday and following Monday in April 2022, allowing for coverage of self-reported GI distress over a period of 7 days during the same training week. During recruitment, the research team was able to question two-thirds (n = 60) of the players, part of one roster (n = 97). To be included, male players needed to be part of the National Collegiate Athletic Association Division I American football team at Arizona State University, in Phoenix, Arizona, USA. Females and visitors at the football facility, and those who were not active members of this American football roster, were excluded from participation. All of the athletes were briefed on the details of this study prior to the completion of informed consent approved by the Arizona State University Institutional Review Board (STUDY00012842). Participants signed informed consent before filling out the questionnaire.

### 2.2. Questionnaire

The questionnaire contained four blocks: the informed consent, general information, dietary information, and the GSRS. The general information included inquiries about the athlete’s demographics including race, ethnicity, football position, and training level. The dietary information contained questions regarding the athlete’s GI symptoms, typical dietary habits, antibiotic use, and over-the-counter and prescription drug use. The GSRS has previously been used to describe GI symptom severity in a wide range of clinical populations, such as patients with irritable bowel syndrome (IBS) [30,31]. The self-administered questionnaire is brief, and has been comprehensively tested for both validity and reliability; moreover, normal values have been made available [32]. The GSRS comprises 15 items, is self-administered, and divided into five clusters of symptoms: reflux, abdominal pain, indigestion, diarrhea, and constipation. These symptoms are graded on a 7-point Likert scale: 1—no discomfort at all, 2—minor discomfort, 3—mild discomfort, 4—moderate discomfort, 5—moderately severe discomfort, 6—severe discomfort, and 7—very severe discomfort. The total time to complete all elements of the questionnaire was approximately 15 min. Once the questionnaires were completed, the principal investigator input the data. After this, another research member checked the quality of the data insertion.

### 2.3. Data Analysis

All of the analyses were conducted utilizing SPSS (v28; SPSS, Inc., Chicago, IL, USA). The data, based on a visual inspection of histograms and assessing skewness and kurtosis, were not normally distributed, and were reported as percentages (%) or as medians and interquartile ranges (IQR). The GSRS outcome averages were reported for the total scores, and for each of the individual items. In addition, the GSRS outcomes were categorized into three main categories based on the 7-point Likert scale as no symptoms (1), moderate symptoms (2–4), and severe symptoms (5–7) [8], in order to better understand the differences between moderate and severe symptom scores. Stratifications were performed for race and/or ethnicity (black or Afro-American vs. white and other races and/or ethnicity), positional differences (skill vs. size positions, as defined in Table 1), nutritional supplement use (i.e., protein supplements, probiotics, and creatine use (yes/no), and NSAID use (yes/no)). No stratifications were performed for subjects reporting food allergies, food omissions, or caffeine supplement use, as the number of athletes surveyed was too low for a reasonable balanced stratification. Non-parametric Mann–Whitney-U tests were used to compare GSRS scores between groups, with a *p*-value ≤ 0.05 set a priori for all analyses. Additionally, eta-squared effect sizes were calculated (η^2^), with 0.01 indicating a small, 0.06 indicating a medium, and ≥0.14 indicating a large effect size.

## 3. Results

### 3.1. Characteristics

Of the 60 athletes who attended lunch to use the dining facility or the football bar for smaller meals during the recruitment period, a total of 76% (n = 46) of all student athletes that were approached provided informed consent. This resulted in 44 complete questionnaires that were analyzed, resulting in a total representation of the roster of 47%. The athletes had an average age of 20.7 ± 1.7 years. Half of the athletes were Afro-American or black (52%, n = 23), followed by white (34%, n = 15) and other races and/or ethnicity (14%, n = 6). A wide range of playing positions was included in this sample, such as lineman, backs and receivers, and special teams, as shown in Table 1, resulting in n = 17 skill and n = 27 size players.

### 3.2. General GI Issues, Food Omissions, Supplements, and Medication

Slightly over half of the athletes (52%, n = 23) reported experiencing GI issues during exercise; within this group, the majority reported 1–2 complaints per week (61%, n = 14), followed by 3–4 times per week (17%, n = 4), and more than 5 times per week (22%, n = 5). A total of 82% (n = 36) of all of the athletes had a bowel movement at least once a day, but within this group, 75% (n = 27) reported a frequency of 2–3 times a day. A total of 11% (n = 5) of the athletes were officially diagnosed with some form of food intolerance or allergy, and 11% (n = 5) reported having experience with eliminating food components from their diet, including dairy (or lactose), gluten, and processed foods. Nutritional supplement use varied between athletes, but the most important supplements related to the main study outcomes were protein supplements (41%, n = 18), creatine (34%, n = 15), probiotics (27%, n = 12), and caffeine-containing supplements including pre-workout (11%, n = 5). Finally, a total of 18% (n = 8) of the athletes reported the regular use of NSAIDs, and 46% (n = 20) of the players reported the use of antibiotics during the last 12 months, of which 14% (n = 6) reported using these less than 3 months prior.

Table 2 displays the calculated average and median outcomes of all scored complaints, the total score of 21.8 ± 10.4, and 17.0 with IQR: 15.0–23.5. The highest average total scores were reported for rumbling (1.80 ± 1.42, and 1.00 with IQR: 1.00–2.00), pain and discomfort in the upper abdomen or pit of the stomach (1.61 ± 0.92, and 1.00 with IQR: 1.00–2.00), and passing gas or flatus (1.61 ± 1.37, and 1.00 with IQR: 1.00–2.00). When comparing athletes who reported the use of protein supplements (41%, n = 18) to those reporting not using these supplements (59%, n = 26), a significantly higher overall GSRS score was reported by those who used additional protein (27.1 ± 12.8, and 22.0 with IQR: 17.0–31.8 vs. 18.1 ± 6.26, and 15.00 with IQR: 15.0–19.3, *p* = 0.001, with a large effect size η^2^ = 0.24). Other significant differences included protein supplement users reporting higher scores for nine out of fifteen items on the GSRS. The greatest differences were reported for diarrhea (2.11 ± 1.53, and 1.50 with IQR: 1.00–3.00 vs. 1.08 ± 0.27, and 1.00 with IQR: 1.00–1.00, *p* = 0.001), passing gas or flatus (2.06 ± 1.51, and 2.00 with IQR: 1.00–2.25 vs. 1.31 ± 1.19, and 1.00 with IQR: 1.00–1.00, *p* = 0.002), and the feeling of an urgent need to use the restroom (2.00 ± 1.72, and 1.00 with IQR: 1.00–3.25 vs. 1.00 ± 0.00, and 1.00 with IQR: 1.00–1.00, *p* = 0.002). There were smaller differences for symptoms including pain and discomfort in the pit of the stomach, rumbling, stomach feeling bloated, loose stools, hard stools, and not completely being able to empty bowels (*p* < 0.037) for protein supplement users vs. non-protein supplement users. The effect sizes (η^2^) for these differences ranged from 0.10–0.22, with most of them considered large. No significant differences were found for the discomfort caused by heartburn, hunger pains, nausea, constipation, and burping (*p* > 0.085).

No differences were reported for GI distress stratifications for race (black or African American vs. other, *p* ≥ 0.08), complaints (NSAIDs users vs. non-users of NSAIDs, *p* ≥ 0.08), playing position (skill vs. line, *p* ≥ 0.156), users of creatine vs. non-users of creatine (*p* ≥ 0.081), and users of probiotics (*p* ≥ 0.101).

**Figure 1:**
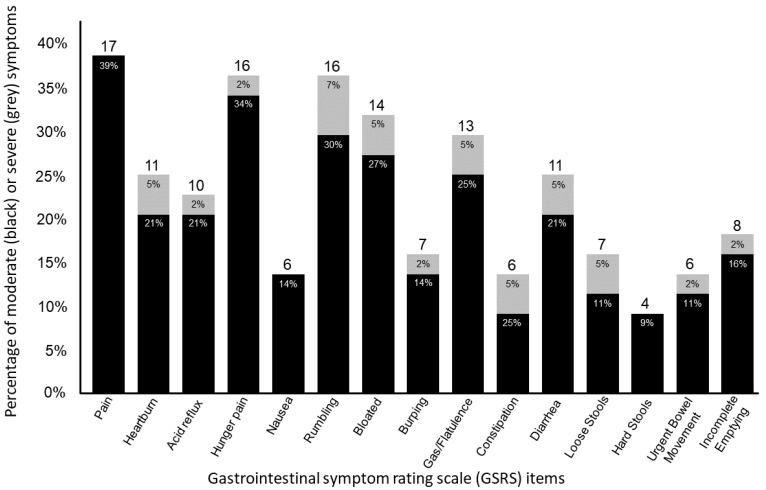
Visual representation of moderate (black) and severe (grey) GSRS complaints displayed as a percentage of reports, with the total number of reports for each symptom at the top of each bar.

When combining the minor to moderate and moderately severe to very severe scores in Figure 1, around one out of three athletes reported pain and discomfort in the upper abdomen or pit of the stomach (39%, n = 17), hunger pain (36%, n = 16), and rumbling (37%, n = 16), of which the latter two also included severe complaints from 2–7% of the athletes.

Of the 44 athletes in this study, 7 athletes (16%) reported at least one symptom as “moderately severe” or worse (GSRS score ≥ 5). Aside from reports of mild to moderate pain and discomfort in the upper abdomen or pit of the stomach, nausea, and hard stool, all other symptoms included 1–3 (2–7%) athletes occasionally reporting more serious complaints. Finally, depending on the symptom, the number of athletes reporting no complaints at all varied from 61% (pain and discomfort in the upper abdomen or pit of the stomach) to 91% (hard stools).

**Figure 2:**
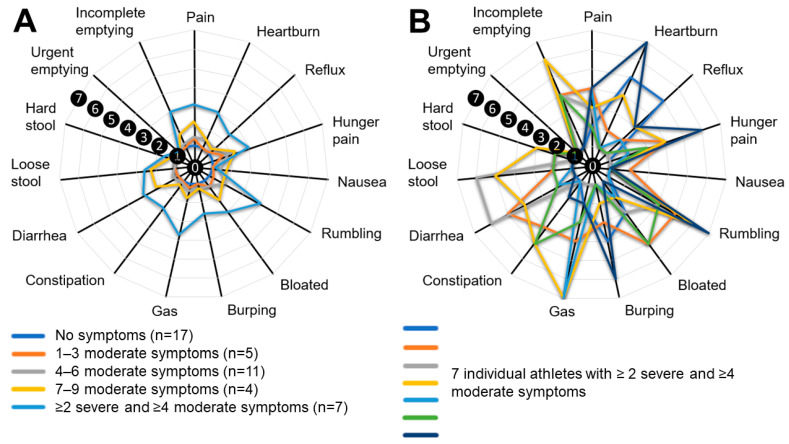
(**A**) Visual representation of mean GSRS symptoms reported as moderate and severe, divided into frequency categories. (**B**) Visual representation of GSRS scores from the seven individuals who reported severe symptoms. Note: scores are indicated by the numbered black circles, indicating no complaints (score 0), up to severe complaints (score 7).

As displayed in Figure 2A, 17 athletes (39%) reported no discomfort for any of the GSRS symptoms, followed by 5 athletes reporting 1–3 moderate symptoms, 11 athletes reporting 4–6 moderate symptoms, and 4 athletes reporting 7–9 moderate symptoms. Finally, the group reporting ≥2 severe symptoms and a wide variety of moderate symptoms (n = 7) reported the highest average score, as indicated by Figure 2A. When isolating this specific group, as shown in Figure 2B, the type and severity reported for different types of symptoms were highly variable between athletes. All the athletes who reported severe complaints, being part of the highlighted group of athletes in Figure 2B, reported a minimum of two severe symptoms, and four or more additional symptoms as moderate.

## 4. Discussion

The aim of this study was to obtain a better exploratory understanding of the self-reported prevalence of GI symptoms and their severity for players of an NCAA Division I level American football team. More than half of the football players in this study reported moderate to severe GI symptom ratings. Although the number of athletes reporting severe complaints was small, the type of reported severe complaints were highly variable between individuals. Additionally, no differences in symptoms experienced were seen when the data were split for race and/or ethnicity, playing position, or NSAID use. Finally, when stratifying for nutritional supplement use, only protein supplementation was associated with a higher prevalence of GI complaints.

Gastrointestinal symptoms experienced by American football athletes are not well documented. Our present findings provide important insight, highlighting the high prevalence in this team sport’s setting. The reporting of 61% overall complaints and 16% moderately severe or worse complaints was much higher than the ~14% reported in a sample that comprised 75% of team-based male athletes [1]. At the same time, the number of complaints in the current study can be considered similar to the 70% reported in elite endurance athletes like marathoners, cyclists, and triathletes [33]. Although the total reporting was lower than the 86% overall complaints in a study with combined team and endurance sports, the 15% moderately severe or worse GI complaints was similar for both studies [8]. Gastrointestinal symptoms can potentially lead to decreased athletic performance or abandonment of competition [34]. This may be concerning for team-based athletes, as this study shows that GI distress in football athletes is similar to that for endurance sports, and may result in similar outcomes.

Gastrointestinal complaints may be the result of impaired gut barrier function, local inflammation, microbiota disbalance, and/or stress-related nutrient malabsorption [2]. The majority of the research around GI symptoms and distress has focused on the occurrence of complaints during moderate to high intensity aerobic exercise [33,35], and there is less information on if and how high-intensity (anaerobic) exercise can lead to an increase in GI symptoms in team sport athletes [1,29]. While speculatory, confounders such as race and/or ethnicity, positional playing differences, food allergies, nutritional supplement use, and NSAID use may contribute to these complaints, we did not find a clear difference in self-reported GI symptoms for most stratifications. This is in line with the inconclusive research available, for example, regarding investigations on the impact of NSAIDS on GI symptoms [1]. In addition, only limited research has looked for a direct relation between protein intake, exercise, and GI symptoms [29]. Participation in high-intensity athletics, such as American football, can increase athletes’ needs for both macro- and micronutrient intake [36]. Although these increased needs can normally be met through increased food intake, athletes may use protein supplements. Both collegiate athletes (66%) [17] and elite athletes (42%) [37] have reported protein supplement use similar to that of the football players in the current study. A reason for this high use of protein supplements is that they can be considered as a convenient method to meet the protein needs for these athletes [38]. The ingestion of large amounts of protein, immediately prior to or during exercise, has been noted to increase feelings of nausea and vomiting in some athletes when compared to the ingestion of carbohydrates [29]; however, the present study did not report higher nausea scores for athletes reporting the use of protein supplementation in comparison to non-users. On the other hand, football players reporting the use of protein supplements reported increased symptom ratings for five out of the fifteen GI complaints, as well as a higher total GSRS score.

The mix of symptoms reported by athletes can be similar to the symptomatology experienced by patients with diagnosed irritable bowel syndrome (IBS) [39], such as bloating, diarrhea, constipation, and abdominal pain [39]. A proper diagnosis to confirm or rule out IBS, which is very common in up to 16% of the US population [39], may be needed to determine the best targeted approach to follow up. The treatment of GI symptoms through mitigation of food intake, regardless of being IBS-related or not, often comes down to the well-informed elimination of products that are rich in certain substances in a trial-and-error fashion, and evaluating the effectiveness. This may include measures such as decreased fiber ingestion, lower fat intake, reduced fructose load, minimizing dehydration, and consuming multiple transporter carbohydrates [40], as well as limiting fructans, excess fructose, lactose, galacto-oligosaccharides, and polyols [41], and avoiding the use of NSAIDs before exercise to reduce the risk for intestinal injury and increased permeability [2]. It is important to emphasize that a small number of athletes in the current study already reported food omissions, but we did not follow up on the actual changes nor on the actual impact of these changes on their diet quality. In the current study, IBS-like symptoms such as pain, bloating, and flatulence were commonly reported, followed by hunger pains, and rumbling of the stomach completing the top five of the most frequently reported complaints. Possible options for treatment to reduce the severity of GI symptoms, including flatulence, urge to defecate, loose stool, and diarrhea in athletes [42], and pain, bloating, nausea, and flatulence in IBS patients [43], involve implementing diets that are low in fermentable oglio- di- and monosaccharides and polyols. The so-called low FODMAP diet suggests the temporary elimination of foods, including dairy (such as milk), onions, wheat-based cereals, beans, and vegetables like artichokes and asparagus [41]. This diet reduced GSRS scores reported by IBS patients significantly for pain, bloating, flatulence [43], as well as reduced daily GI symptoms in healthy runners who reported exercise-induced GI symptoms [42].

The following practical suggestions can be considered for each of the frequently reported symptoms by American football athletes in this study: pain in the pit of the stomach that often develops directly after eating [44] may be provoked by milk and gluten-containing foods [45]. Pain originating in the stomach can be a symptom of lactose intolerance, gluten intolerance, and celiac disease [46,47,48]. Most commonly, when diagnosed with a specific intolerance, the solution is to eliminate the foods triggering the intolerance [47,48]. Bloating may likely result from multiple causes, such as carbonated drinks [49] and a high fiber intake [49,50]. In addition, limiting fat and sugar intake, as well as the use of probiotics and antibiotics (such as neomycin or rifaximin), have been suggested to reduce bloating in IBS patients [49]. Flatulence has been associated with diets rich in fermentable residues [51]; therefore, avoiding meals including cereals made from wheat or corn, bananas, peaches, broccoli, and cabbage, may reduce these complaints [52]. Treatment approaches for hunger pain are not well supported by the literature; however, the sensation of hunger and a high gastric emptying rate have been correlated [53,54]. Practical advice includes eating smaller meals that are high in protein [55] at multiple times during the day, as postprandial ghrelin levels are reported to decrease for approximately 90 min; after this, the levels progressively increase [56]. At the same time, a diet rich in high-protein foods may reduce the need for the use of protein supplements. This may be favorable, as the use of protein supplements in the current study was associated with a higher incidence of GI distress. Rumbling of the stomach occurs during digestion caused by peristaltic movements [57]. No treatments for this symptom have been described in the literature; however, considering the knowledge about low GI tract contents, one possible method is to eat multiple, smaller meals throughout the day, which may help to serve as a buffer to reduce these sounds.

A strength of this study is that it provides unique insights into the prevalence of GI symptoms and their severity in American football athletes; until now, this has been underrepresented in the literature. No information on dietary intake, specifics on supplement use frequency or dosage, and markers of gut injury was collected in this research. As a result of this, no direct causation can be drawn between their symptoms and the potential underlying pathologies of these symptoms. Furthermore, the sample size was small, in an all-male population, with the sampling conducted during a training phase in preparation for the regular competitive season. Therefore, we suggest that well-controlled future research should further investigate the causality of the relations found between the GSRS scores and protein supplementation and/or protein intake in general. At the same time, the GSRS was developed to phenotype the pattern of complaints for specific populations [31,58]; therefore, these data should cautiously be used because of the relatively small sample size of athletes included, as a first reference for sports health professionals (such as sports dietitians, athletic trainers, and strength and conditioning coaches) to identify the types of symptoms and associated factors that collegiate American football players may experience.

## 5. Conclusions

A substantial number of collegiate DI American football athletes sampled in this study reported GI distress that was similar to that reported in IBS patients. Overall, the athletes reported a high variability in the types of complaints, suggesting the importance of an individualized follow-up to determine the etiology and potential treatment of these complaints. By association, dietary factors such as the use of protein supplements may impact the severity of reported complaints, but a proper well-controlled investigation is needed to support the causality of this relation found in the current study.

## Figures and Tables

**Table 1:** Demographics of the football players (n = 44) in this study.

	Number or Percentage
**Age (years)**	20.7 ± 1.7
**Race**	
Afro-American or black	52% (n = 23)
White	34% (n = 15)
Other	14% (n = 6)
**Playing position**	
**Size**	
Defensive Back	14% (n = 6)
Defensive-Line	11% (n = 5)
Line Backer	16% (n = 7)
Offensive-Line	20% (n = 9)
**Skill**	
Kicker/Punter	7% (n = 3)
Quarter Back	5% (n = 2)
Running Back	9% (n = 4)
Tight End	2% (n = 1)
Wide Receiver	16% (n = 7)
**Use of medication**	
Use of non-steroidal anti-inflammatory drugs	18% (n = 8)
Use of antibiotics in the last 12 months	46% (n = 20)
**Bowel movement and complaints**	
Passing bowel movement at least once a day	82% (n = 36)
Experience of gastrointestinal issues during exercise	52% (n = 23)
Diagnosed with food intolerance of allergy	11% (n = 5)
Tried to eliminate any dietary components	11% (n = 5)
**Self-reported use of frequently reported dietary supplements**	
Protein and amino acids	41% (n = 18)
Creatine	34% (n = 15)
Probiotics	27% (n = 12)
Caffeine	11% (n = 5)

**Table 2:** Outcomes for the discomfort based on the validated gastrointestinal symptoms rating scale (GSRS), and stratified outcomes for American football athletes (n = 44) using protein supplements or not reported as mean ± SD and median (IQR), P-value and effect size Eta-squared (η^2^).

Sensations during the Last Week	All (n = 44)	Using Protein Supplements (n = 18)	Not Using Protein Supplements (n = 26)	*p*-Value and (η^2^).
Total Score	21.8 ± 10.4 17.0 (15.0–23.5)	27.1 ± 12.8 22.0 (17.0–31.8)	18.1 ± 6.26 15.0 (15.0–19.3)	0.001 * (0.24)
Pain or discomfort in upper abdomen/stomach pit	1.61 ± 0.92 1.00 (1.00–2.00)	1.94 ± 0.94 2.00 (1.00–3.00)	1.39 ± 0.85 1.00 (1.00–1.25)	0.015 * (0.14)
Heartburn	1.48 ± 1.17 1.00 (1.00–1.75)	1.61 ± 1.15 1.00 (1.00–2.00)	1.39 ± 1.20 1.00 (1.00–1.00)	0.283 (0.03)
Acid reflux	1.34 ± 0.78 0.00 (1.00–1.00)	1.61 ± 1.04 1.00 (1.00–2.00)	1.15 ± 0.46 1.00 (1.00–1.00)	0.037 * (0.10)
Hunger pains	1.73 ± 1.19 1.00 (1.00–2.00)	1.89 ± 1.18 1.00 (1.00–3.00)	1.61 ± 1.20 1.00 (1.00–2.00)	0.324 (0.02)
Nausea	1.18 ± 0.50 0.00 (1.00–1.00)	1.28 ± 0.57 1.00 (1.00–1.25)	1.12 ± 0.43 1.00 (1.00–1.00)	0.186 (0.04)
Rumbling	1.80 ± 1.42 1.00 (1.00–2.00)	2.27 ± 1.56 2.00 (1.00–3.25)	1.46 ± 1.24 1.00 (1.00–1.25)	0.021 * (0.12)
Stomach feeling bloated	1.57 ± 1.04 1.00 (1.00–2.00)	2.11 ± 1.41 1.50 (1.00–3.00)	1.19 ± 0.40 1.00 (1.00–1.00)	0.020 * (0.15)
Burping	1.34 ± 0.96 0.00 (1.00–1.00)	1.50 ± 0.92 1.00 (1.00–2.00)	1.23 ± 0.99 1.00 (1.00–1.00)	0.085 (0.07)
Passing gas or flatus	1.61 ± 1.37 1.00 (1.00–2.00)	2.06 ± 1.51 2.00 (1.00–2.25)	1.31 ± 1.19 1.00 (1.00–1.00)	0.002 * (0.22)
Constipation	1.32 ± 0.96 0.00 (1.00–1.00)	1.67 ± 1.41 1.00 (1.00–1.25)	1.08 ± 0.27 1.00 (1.00–1.00)	0.139 (0.05)
Diarrhea	1.50 ± 1.11 1.00 (1.00–1.75)	2.11 ± 1.53 1.50 (1.00–3.00)	1.08 ± 0.27 1.00 (1.00–1.00)	0.001 * (0.25)
Loose stools	1.36 ± 1.06 0.00 (1.00–1.00)	1.83 ± 1.54 1.00 (1.00–2.00)	1.04 ± 0.196 1.00 (1.00–1.00)	0.008 * (0.16)
Hard stools	1.11 ± 0.39 0.00 (1.00–1.00)	1.28 ± 1.58 1.00 (1.00–1.25)	1.00 ± 0.00 1.00 (1.00–1.00)	0.013 * (0.14)
Urgent need to have a bowel movement	1.41 ± 1.19 0.00 (1.00–1.00)	2.00 ± 1.72 1.00 (1.00–3.25)	1.00 ± 0.00 1.00 (1.00–1.00)	0.002 * (0.23)
Not completely emptying bowels	1.43 ± 1.09 0.00 (1.00–1.00)	1.94 ± 1.55 1.00 (1.00–3.25)	1.08 ± 0.27 1.00 (1.00–1.00)	0.021 * (0.12)

A *p*-value is considered significant ≤0.05, and indicated by *, and effect size eta squared is indicated as η^2^.

## Data Availability

Not applicable.
